# Management of Dentin Hypersensitivity by National Dental Practice-Based Research Network practitioners: results from a questionnaire administered prior to initiation of a clinical study on this topic

**DOI:** 10.1186/s12903-017-0334-0

**Published:** 2017-01-13

**Authors:** Dorota T. Kopycka-Kedzierawski, Cyril Meyerowitz, Mark S. Litaker, Sidney Chonowski, Marc W. Heft, Valeria V. Gordan, Robin L. Yardic, Theresa E. Madden, Stephanie C. Reyes, Gregg H. Gilbert, National Dental PBRN Collaborative Group

**Affiliations:** 1 Eastman Institute for Oral Health, University of Rochester, 625 Elmwood Ave, Rochester, NY 14620 USA; 2 University of Alabama at Birmingham, Birmingham, AL USA; 3 Private practice of general dentistry, Morristown, NJ USA; 4 University of Florida, Gainesville, FL USA; 5 Health Partners, Minneapolis, MN USA; 6 Private practice of periodontics, Olympia, WA USA; 7 University of Texas Health Science Center at San Antonio, San Antonio, TX USA

**Keywords:** Dentin hypersensitivity, National Dental Practice-Based Research Network

## Abstract

**Background:**

Dentin hypersensitivity (DH) is a common problem encountered in clinical practice. The purpose of this study was to identify the management approaches for DH among United States dentists.

**Methods:**

One hundred eighty five National Dental Practice-Based Research Network clinicians completed a questionnaire regarding their preferred methods to diagnose and manage DH in the practice setting, and their beliefs about DH predisposing factors.

**Results:**

Almost all dentists (99%) reported using more than one method to diagnose DH. Most frequently, they reported using spontaneous patient reports coupled with excluding other causes of oral pain by direct clinical examination (48%); followed by applying an air blast (26%), applying cold water (12%), and obtaining patient reports after dentist’s query (6%). In managing DH, the most frequent first choice was desensitizing, over-the-counter (OTC), potassium nitrate toothpaste (48%), followed by fluorides (38%), and glutaraldehyde/HEMA (3%). A total of 86% of respondents reported using a combination of products when treating DH, most frequently using fluoride varnish and desensitizing OTC potassium nitrate toothpaste (70%). The most frequent predisposing factor leading to DH, as reported by the practitioners, was recessed gingiva (66%), followed by abrasion, erosion, abfraction/attrition lesions (59%) and bruxism (32%).

**Conclusions:**

The majority of network practitioners use multiple methods to diagnose and manage DH. Desensitizing OTC potassium nitrate toothpaste and fluoride formulations are the most widely used products to manage DH in dental practice setting.

## Background

Dentin hypersensitivity (DH) has been defined as a “short, sharp pain arising from exposed dentin in response to stimuli, typically thermal, evaporative, tactile, osmotic or chemical, and that cannot be ascribed to any other form of dental defect or pathology” [1]. One of the most frequent patient complaints is related to cold stimuli, although pain may also occur when consuming acidic foods (mainly fruit), sweets and salty foods. Tactile stimulus provocation frequently occurs when patients brush their teeth or rub the sensitive area with a finger nail [2].

DH is a relatively common problem encountered in clinical practice. It may disturb patients during eating, drinking and brushing. The prevalence of DH has been reported to be in the range of 8 to 57% [1, 3–11]. The wide range could be related to different methods used to diagnose this condition and whether prevalence was assessed by clinical examination and/or questionnaires [12].

For example, among 780 patients from the Health Examination Center of National Taiwan University Hospital, the prevalence of DH was 32% [9]. The self-reported prevalence of DH among regular attenders in three general dental practices in the United Kingdom was 52%; DH was most prevalent among 30–40 year old patients and it was more common among female patients [10]. A cross-sectional study conducted by 19 dental practitioners in the United Kingdom examined 4841 patients in one calendar month and found that 4.1% of patients were diagnosed with DH based on the dentist examination. Upper premolars were the most commonly affected, and cold drinks initiated DH most often. More sensitive teeth were found in patients with periodontal disease who also smoked [13].

Based on a cross-sectional survey of 787 adult patients from 37 general dental practices within the Northwest PRECEDENT Practice Based-Research Network (PBRN), the prevalence of DH was 12.3% [11]. Patients with hypersensitivity had, on average, 3.5 hypersensitive teeth. The prevalence was highest among patients who were 18–44 years of age and lowest among patients who were 65 years of age or older. The prevalence was higher among women, patients with gingival recession and patients who used at home tooth whitening products [11].

No clear consensus among Northwest PRECEDENT dentists existed for successfully treating DH, but fluoride varnishes and gels apparently were most widely employed. Dentists also expressed high levels of interest in testing fluoride varnishes and gels, as well as glutaraldehyde/HEMA and restorative treatments, in future studies [14].

DH affects patients of any age with its peak occurrence in middle-aged adults. It may affect any tooth, but most often affects canines and first premolars, probably because they are prominent in the arch and they are exposed to higher pressure during tooth brushing. It may present clinically on any tooth surface, but most often occurs on the buccal cervical margins of teeth. Several theories of DH have been proposed. These include hydrodynamic, odontoblast transduced mechanism and direct innervation theories [7]. None of these mechanisms fully explain this phenomenon. Although dentin sensitivity appears to be prevalent, no universally used or highly reliable desensitizing agents or treatment modalities have been identified [15, 16].

Recently the Practitioners Engaged in Applied Research and Learning Network (PEARL Network) conducted a randomized clinical trial in the practice setting to assess the outcomes of noncarious cervical lesion treatment choices [17]. The overall objective was to determine the efficacy of three randomly assigned treatments for hypersensitive noncarious lesions: chemoactive dentifrice use, dentin bonding agent with sealing and flowable resin-based composite restoration. The secondary outcomes were tubule occlusion, retention of resin coating, retention of restoration and change in lesion size. Results suggest that placement of the sealant or resin restoration was effective in reducing hypersensitive noncarious cervical lesions over the 6-month study period.

According to a survey of dental practitioners conducted by the Canadian Advisory Board on Dentin Hypersensitivity, approximately 50% of the respondents reported lack of confidence in managing patients’ pain due to DH [1]. The Canadian Advisory Board on Dentin Hypersensitivity suggested that providers initiate management of this condition by applying desensitizing treatment that is noninvasive; i.e., desensitizing toothpaste and/or topical agents. Some dental providers use a stepped approach to treatment with multiple visits; others apply and prescribe multiple treatments at one time. Invasive treatments of DH are also performed by placing a restoration on an otherwise healthy tooth [1].

Although DH has been studied previously in the practice-based research setting, there have been wide differences among clinicians as to the methods used to diagnose and manage DH; furthermore the prior data were constrained to one region of the US. Therefore, the purpose of this study was to identify in a broader national context the preferred methods to diagnose and manage DH in the practice setting and to assess practitioners’ beliefs about DH predisposing factors. In addition, we assessed whether practitioner and practice characteristics were associated with practitioners’ selected treatments and approaches to care.

## Methods

As an initial phase of a prospective, multicenter cohort study of patients with DH, 185 National Dental Practice-Based Research Network (National Dental PBRN) clinicians answered an online questionnaire related to the diagnostic methods, treatment modalities and predisposing factors of DH. The questionnaire is publicly available and it is enclosed in a supplementary file with the manuscript [18]. The current paper reports the results of the online questionnaire that was administered prior to initiation of a cohort study on this topic. The network is a consortium of dental practices and organizations that participate in clinical research studies and comprises six regions across the US [18, 19].

Initially, the study investigators pilot tested the questionnaire with six practitioners to assess its length, acceptability, and internet browser compatibility. Based on practitioner feedback, the study investigators administered a revised questionnaire to 24 additional practitioners to quantify test-retest reliability for 94 items (text items were not evaluated). Agreement between responses was calculated for each of 24 practitioners. For each practitioner, if there was no response for an item at both test and retest, this item was not included in the evaluation. Percentage agreement was calculated as the number of items for which the test and retest responses were the same, divided by the number of items for which the practitioner provided responses, multiplied by 100. Descriptive statistics were calculated for the practitioner-level agreement values. The mean number of items for which responses were provided was 37.63, minimum 19, maximum 48. The mean number of items showing agreement was 24.33, minimum 13, maximum 33. The mean agreement across the 24 practitioners was 65.01%. The minimum and maximum agreement for individual practitioners was 45.65 and 100%. The questionnaire was not modified after testing. The test-retest questionnaires were completed in February 2015. Practitioners also complete an enrollment questionnaire that describes characteristics about themselves and their practice(s). Selected questions from the enrollment questionnaire were used to explore which characteristics were associated with practitioners’ treatments and approaches to DH care. Practitioner variables included: age, gender, race/ethnicity and dental specialty. Practice variables included: practice size, location and practice type.

The study participants were invited to enroll in the study in March 2015. Any National Dental PBRN practitioner (i.e., general dentist and specialist) who was enrolled in the network at the full participation level was eligible to participate in the study. One hundred eighty five practitioners were study-ready by the end of July 2015, having completed all necessary human subjects and conflict of interest training as required by the National Dental PBRN procedures.

### Statistical Analysis

Sample size considerations were based on precision of estimation of percentages, represented by the widths of 95% confidence intervals adjusted for the effect of clustered sampling due to enrolment of multiple patients per dental practice. Adjustment for clustering used variance inflation factors calculated for a range of likely values of intracluster correlation (ICC). Power to detect a difference between proportions of dentists using each of the treatment modalities was estimated based on cluster-adjusted chi-square tests to approximate the power of the proposed GEE analysis. Based on this analysis, the target sample size of the study was set at 180 practitioners.

Descriptive statistics including frequencies, means, medians, standard deviations and quartiles were calculated. The chi-square and Fisher’s exact tests were used to compare distributions of categorical variables. Analysis of variance and the Kruskal-Wallis test were used for the analysis of continuous variables. The Tukey and Wilcoxon rank sums tests were used for post-hoc comparisons. The analysis was conducted using SAS^®^ Release 9.4 statistical software. *P*-values less than 0.05 were considered statistically significant.

## Results

### Practitioners’ characteristics, practice location and practice type

Among the 185 practitioners who completed the questionnaire, 34 represented the Western region, 29 represented the Midwest region, 30 represented the Southwest region, 30 represented the South Central region and 31 practitioners each represented the South Atlantic and Northeast regions of the National Dental PBRN. Table 1 summarizes practitioners’ characteristics, practice location and type. One hundred nineteen practitioners were male (64%) and 66 (36%) were female. The majority (79%) identified themselves as White, 4% as African-American, 10% as Asian and 7% as other racial category. Practitioners’ ages differed significantly by network region (*p* = 0.02). Practitioners in the Northeast and the South Central regions were the oldest (mean (SD) age of 56.1 (11.15) years) and practitioners from the South Atlantic region were the youngest (mean (SD) age of 48.7 (12.17) years; *p* = 0.02, ANOVA).

**Table 1:** Characteristics of participating practitioners and their practice(s) (*N* = 185)

Practitioner and practice characteristics	
Gender	N (%)
Male	119 (64)
Female	66 (36)
Race	N (%)
White	146 (79)
African-American	8(4)
Asian	18 (10)
Other	12 (7)
Age	Years (SD)
Mean	52 (11.4)
Median	55
Range	27–58
Practice location	%
Inner City of Urban Area	9
Urban Area	27
Suburban	50
Rural	14
Practice type	%
Owner of a private practice	73
Associate/employee of a private practice	10
Health Partners Dental Group	4
Permanente Dental Associates	7
Other managed care/preferred provider	1
Public health practice	3
Dental School/academic institution	2
Specialty	N (%)
General Dentist	173 (94)
Specialist	12 (6)

### Diagnosis of Dentin Hypersensitivity

Table 2 summarizes the most frequent methods practitioners reported using when diagnosing DH. Spontaneous patient report confirmed by the dental examination, was chosen most frequently as the first choice (48%). This was followed by applying air blast (26%), scratching dentin with a dental explorer (12%), patient report after dentist’s query (6%), using other methods, most likely applying endo-ice (4%), and applying cold water (2%).

**Table 2:** Most frequent choices used when diagnosing DH (*N* = 182)

Most frequent methods used when diagnosing DH	N (%)
Spontaneous patient report confirmed by the dental exam	88 (48)
Applying air blast	47 (26)
Scratching dentin with dental explorer	22 (12)
Obtaining patient report after dentist’s query	11 (6
Other (most likely using endo ice)	8 (4)
Applying cold water	4 (2)
Requesting numeric rating of pain	2 (1)

### Treatment Modalities

As shown in Table 3, the practitioners reported using multiple products when managing DH. Almost all practitioners (97%) reported routine use of fluoride formulations, followed by desensitizing over-the-counter (OTC) potassium nitrate toothpaste (94%). Glutaraldehyde/HEMA products were reported as being used routinely by 42% participating in the survey. Interestingly, bonding agents and restorative treatments were reported to be used routinely respectively by 52 and 64% of the practitioners when treating DH.

**Table 3:** Treatment modalities routinely used when treating Dentin Hypersensitivity (Practitioners had options to check multiple answers)

Treatment modality	N (%)
Fluoride formulations (gels, varnishes, pasted and rinses)	180 (97)
Desensitizing over-the counter (OTC) potassium nitrate toothpastes	173 (94)
Glutaraldehyde/HEMA products	78 (42)
Bonding agents	97 (52)
Sealants	30 (16)
Restorative treatments	119 (64)
Lasers	6 (3)
Oxalates	21 (11)
Advice	41 (22)
Other	35 (19)

As shown in Table 4, practitioners reported that the most frequent, practitioner reported, first choice of products used when managing DH was: OTC potassium nitrate toothpaste (48%), followed by fluoride formulations (38%) and glutaraldehyde/HEMA products (3%). Four percent of the practitioners reported giving advice (i.e., related to diet and dental habits) to their patients as their first choice of treatment modality. A total of 86% of the respondents reported using a combination of products when treating DH, most frequently fluoride varnish and desensitizing OTC potassium nitrate toothpaste (70%).

**Table 4:** Most frequent products used when managing DH (these were first choices of products indicated by the practitioners)

Most frequent products used when managing DH	N (%)
OTC potassium nitrate toothpaste	88 (48)
Fluoride formulations	70 (38)
Giving advice (related to diet and dental habits)	7 (4)
Other	7 (4)
Glutaraldehyde/HEMA	6 (3)
No treatment	2 (1)
Restorative treatments	1 (2)
Bonding agents	1 (.5)
Lasers	1 (.5)
Oxalates	1 (.5)

### Predisposing Factors

As summarized in Table 5, practitioners indicated their first choice of potential factors that may be related to dentin hypersensitivity. Recessed gingiva was chosen by 66% of the practitioners, followed by abrasion, erosion, abfraction and/or attrition lesions (59%). Thirty two percent indicated that bruxism contributes to DH and that it was their first choice of predisposing factors. Excessive tooth whitening and frequent consumption of citric juices and/or carbonated drinks were chosen by 17 and 15% of practitioners, respectively, as first choices for predisposing factors of DH.

**Table 5:** Most frequent predisposing factors of DH as indicated by the practitioners (Practitioners indicated their first choice of predisposing factors)

Predisposing factor	N (%)
Recessed gingiva	122 (66)
Abrasion, erosion, abfraction and/or attrition lesions	109 (59)
Bruxism	59 (32)
Excessive tooth whitening	31 (17)
Frequent consumption of citrus juices and/or carbonated drinks	28 (15)

### Diagnosis, management and predisposing factors, by network region and practitioners’ characteristics

Practitioners’ diagnostic and management methods did not differ significantly across the six network regions. There were no age differences in diagnostic methods, except for using “other methods” to diagnose DH (*p* < 0.0001, Fisher’s exact test). Only 2% of practitioners in the younger age category (younger than 55 years) indicated using “other methods”, compared to 20% in the older age category (55 years of age and older). Most of the responses in the “other” category suggested using soft bristle toothbrushes, recommending gingival grafting, occlusal adjustments, and fabricating occlusal guards.

There were no practitioner gender differences in diagnostic methods, except when using an explorer (*p* = 0.015, Fisher’s exact test). More male dentists than female dentists use a dental explorer to diagnose DH (84 versus 68%).

There were no regional differences in dentists’ beliefs regarding predisposing factors to DH, except for bruxism (*p* = 0.047, chi-square test). The highest percentage of practitioners from the South Atlantic region (87%) had chosen bruxism as one of the predisposing factors of DH; the lowest percentage was chosen by practitioners from the Western region (53%).

The practice locations did not differ significantly across the 6 regions, however the practice type did differ significantly by region (*p* < 0.0001, chi-square test). Almost 87% of practitioners from the Southwest and the South Central regions identified themselves as owners of a private practice, 83% practitioners from the South Atlantic region, 74% practitioners from the Northeast region, 59% practitioners from the Western region and 52% of practitioners from the Midwest region.

## Discussion

These results suggest that when diagnosing DH practitioners most frequently rely on spontaneous patient report, confirmed by the dental examination, followed by applying an air blast and scratching dentin with a dental explorer. Our finding is consistent with the 2008–2009 study by Northwest PRECEDENT practitioners, which suggested that the most frequently reported diagnostic method was spontaneous patient report [14]. Patient reports, prompted by a query from the dentist, were also common, but used less frequently. Additionally, dentists employed a dental explorer or air blast to assess DH [14]. Our findings indicate that practitioners confirm their patients’ reports with a dental examination to diagnose DH. The diagnosis of DH is thus made by excluding other oral conditions that may explain pain and discomfort in the oral cavity.

These findings also suggest that fluoride formulations and OTC potassium nitrate toothpastes were the most frequent products used to treat DH. Almost 97% of practitioners reported routinely using fluoride formulations and 94% reported routinely using OTC potassium nitrate toothpastes. Almost half (48%) used OTC potassium nitrate toothpaste and 38% used fluoride formulations as their first choice when treating DH. This finding is consistent with the PRECEDENT study, wherein dentists reported using fluoride formulations most commonly and it was the only treatment modality used by more than 50% of respondents. Almost half the PRECEDENT dentists (47%) reported using OTC potassium nitrate toothpastes when managing DH [14]. Our findings also suggest that 86% of respondents used a combination of products when treating DH, most frequently using fluoride varnish and desensitizing OTC potassium nitrate toothpaste (70%); suggesting that most practitioners combine in-office treatment with at-home treatment.

When reporting predisposing factors of DH, 66% of practitioners reported that recessed gingiva was their first choice, followed by abrasion, erosion, abfraction and/or attrition lesions (59%) and bruxism (32%). This finding is supported by the most accepted theory related to DH, the hydrodynamic theory, which proposes that stimuli (thermal, physical or osmotic changes) cause displacement of the fluid that exists within the dentinal tubules and this mechanical disturbance activates the nerve endings in the pulp [20]. This requires that the dentin must be exposed to the oral cavity. In addition to using fluoride varnish and desensitizing OTC potassium nitrate toothpaste, the majority of practitioners indicated restorative treatments when managing DH, most likely restoring abfraction lesions. Older practitioners were more likely than younger practitioners to report occlusal adjustments and fabricating occlusal guards as “other” treatment when managing DH.

Regarding practitioner and practice characteristics that are associated with managing DH, our results suggest that younger and older dentists use similar methods when diagnosing DH. More male practitioners than female practitioners reported using a dental explorer when diagnosing DH. Practitioners 55 years of age or older more often suggested gingival grafting, occlusal adjustments and fabrication of occlusal guards when indicating treatment options used for DH. One of the possible explanations that older practitioners suggested more options when managing DH could be that they had more experience in managing DH than younger practitioners. There are regional differences in beliefs regarding predisposing factors for DH. Most practitioners from the South Atlantic region (87%) indicated bruxism as one of the predisposing factors, while only 53% dentists from the Western region reported this.

Practitioners reported using similar methods when diagnosing and managing DH in their offices, regardless of their practice location, practice type, and network region. As mentioned above, there were a few differences in diagnostic methods and treatment options offered when comparing younger to older practitioners and male to female practitioners; however, the differences were not significantly different between the six network regions.

This study does have some limitations; and interpretation of its conclusions should take these into account. This study relied on questionnaire data rather than direct observation of clinical procedures. Although network practitioners have much in common with dentists at large, it is possible that their reports on diagnosis and treatment of DH and their beliefs about DH predisposing factors are not representative of dentists at large [21, 22]. Additionally, network members are not recruited randomly; their participation in the network (*e.g.,* an interest in participating in clinical research studies) may make them unrepresentative of dentists at large. While we cannot assert that network dentists are entirely representative of US dentists, we can state that they have much in common with dentists at large, while also offering substantial diversity in these characteristics. This assertion is warranted because: 1) substantial percentages of network dentists are represented in the various response categories of the characteristics in the Enrollment Questionnaire; 2) findings from several network studies document that network dentists report patterns of diagnosis and treatment that are similar to patterns determined from non-network dentists [23–25] and 3) the similarity of network dentists to non-network dentists using the best available national source, the 2010 ADA Survey of Dental Practice [26].

## Conclusions

The majority of network practitioners use multiple methods to diagnose and manage DH. Desensitizing OTC potassium nitrate toothpaste and fluoride formulations are the most widely reported products used to manage DH in the practice setting. The majority reported that recessed gingiva, followed by the abrasion/erosion; abfraction/attrition lesions and bruxism most likely contribute to DH.

## Abbreviations

MDH: Management of dentin hypersensitivity
National Dental PBRN: National Dental Practice-Based Research Network
